# Depression vulnerability involves brain activity and connectivity changes consistent with cholinergic deviancy

**DOI:** 10.1016/j.nicl.2025.103941

**Published:** 2025-12-31

**Authors:** Peter Stiers, Zoe Samara, Kyran J.R. Kuijpers, Elisabeth A.T. Evers, Johannes G. Ramaekers

**Affiliations:** Department of Neuropsychology and Psychopharmacology, Maastricht University, 6229 ER Maastricht, The Netherlands

**Keywords:** Major depression disorder, Face perception, Task-related fMRI, Resting-state functional connectivity, Visual attention network, Acetylcholine

## Abstract

Behavioral and imaging studies suggests that emotional biases in the perception of faces associated with major depression disorder (MD) may be embedded within a broader sensory processing deficit. Increased cortical acetylcholine in MD suggest that this deficit may be related to abnormal attention modulation of sensory areas. It is not clear, however, whether these problems are a manifestation of the disease or whether they precede symptom onset. To investigate this, we applied functional magnetic resonance imaging (fMRI) to look for brain activity changes that participants with a family risk of MD (N = 30) shared with participant with MD (N = 28), compared to matched controls (N = 28). Participants were scanned while performing gender categorization of sad, happy, and neutral face pictures, as well as during a state of rest. Task-related activity changes, shared by participants at risk of and suffering from MD, were mostly seen in the posterior brain: increased activity in dorsal attention and visual association cortex, and decreased in lower visual areas. The changes did not differ between neutral faces and faces expressing an emotion. The at risk and MD participants additionally showed increased functional connectivity between the dorsal attention clusters and the lingual gyrus, and decreased connectivity with the lateral occipital complex (LOC). Lastly, they also had in common increased functional connectivity of magnocellular basal forebrain seeds with LOC and visual association cortex. These changes are consistent with an acetylcholine-mediated change in attention-guided sensory processing of all environmental events, which is discernable even before the first MD episode.

## Highlights

• Depression vulnerability markers are shared by patients and their family members.
• fMRI markers show changed dorsal attention activity during face perception.
• Changes are consistent with acetylcholine-mediated moderation of top-down influences.
• These changes are seen in family members with no own history of depression.
• Vulnerability for depression involves general changes in neural processing of events.

## 1 Introduction

Major Depressive Disorder (MD) is the leading cause of disability worldwide (World Health Organization, 2017). On average, one in five people will experience MD in their lifetime (Bromet et al., 2011), more than once in more than half of them (average estimates range from five to nine) (Barreraet al., 2007, Boland and Keller, 2009, Burcusa and Iacono, 2007, Kessler and Walters, 1998). The disease impact is debilitating for the patients, their environment, and societal support systems (Burcusa and Iacono, 2007, Malhi and Mann, 2018). This impact could be drastically reduced by efficient prevention and early intervention, particularly in MD-susceptible populations. The strongest and most reliable risk factor for the development of MD is having a family history of the disorder (Gotlibet al., 2014, Talatiet al., 2013). First-degree relatives of MD patients have a three-fold increased risk of developing the disease (Sullivanet al., 2000, Williamsonet al., 2004). Identifying objective features of MD vulnerability in this population of individuals with a family risk would be of great importance in the development of prevention and early intervention programs.

Studying people at risk of MD is important for another reason: it provides a window on potentially predisposing features of MD, which may play a role in the pathophysiology of the disease. Deviant characteristics that people at risk for MD share with patients with MD can not be seen as mere manifestations of the MD state. Instead, they are potential vulnerability markers, pointing to disposing conditions that may lead to MD under specific circumstances. For instance, several studies have indicated that people with family risk for MD share emotional biases with people with MD, such as in the perception of emotional expressions (Dearing and Gotlib, 2009, Joormannet al., 2010, Joormannet al., 2007, Lopez-Duranet al., 2013) or information about the self (Kohler et al., 2011). This suggests that certain emotional predispositions may play a role in the development of MD and are not merely consequences of the MD state. In line with this suggestion, neuroimaging markers of MD related to the processing of emotional content of facial expressions have been found to predict responsiveness to antidepressant medication (Williams et al., 2015) and risk of relapse to depression (Kudinova et al., 2016). This suggests that the processing of affect in faces may tap into the central aspects of MD dysfunction.

Several lines of research suggest that the affective processing deficit that is commonly associated with MD, is embedded in a broader problem with the processing of sensory information. Firstly, people with MD are frequently reported to perform impaired on tasks with non-emotional stimuli, ranging from typical cognitive tasks focusing on working memory (Mannieet al., 2010, Nordet al., 2020, Watterset al., 2019), response inhibition (Hoffmannet al., 2019, Watterset al., 2019), or visuospatial attention (Cavezianet al., 2007, Pardoet al., 2006), to visual-perceptual tasks assessing motion coherence sensitivity (Nortonet al., 2016, Songet al., 2021), contrast sensitivity (Bublet al., 2009, Famet al., 2013), or binocular rivalry (Jiaet al., 2020, Jiaet al., 2015).

Secondly, task-related brain activation studies in patients with MD frequently observe deviant activity in the posterior brain (Chenet al., 2019, Fitzgerald, 2013, Grahamet al., 2013, Groenewoldet al., 2013). Although these activity differences have received little attention in MD models, they are backed-up by several reports of structural grey matter reduction in the visual/attentional cortex of patients with MD—i.e. parahippocampal, fusiform and inferior temporal gyri, intraparietal cortex (Foland-Rosset al., 2015, Ozalayet al., 2016, Papmeyeret al., 2015, Suhet al., 2019). In addition to these structural abnormalities in the posterior brain, structural changes are also seen in the frontal brain, associated with cognition (insula, dorsolateral prefrontal cortex, precentral gyri) (Amicoet al., 2011, Opelet al., 2016, Schmaalet al., 2017) and affect processing (orbital, and medial frontal cortex) (Opelet al., 2016, Ozalayet al., 2016, Schmaalet al., 2017).

Thirdly, several studies reported MD-related alterations in the cerebral level of the neurotransmitter acetylcholine (ACh). ACh modulates sensory information processing by optimizing the attentional optimization of sensory brain networks, leading to improved stimulus detection contingent upon task requirements (Bentleyet al., 2011, Demeter and Sarter, 2013). Cerebral ACh release originates from neurons in the magnocellular basal forebrain (MBF), mostly in the basal nucleus of Meynert (cell group ch4) and to a lesser extend in the medial cell groups (ch1-3). These cholinergic cell groups are under control of neural circuits involved in regulation of attention and cognitive effort, such as the anterior insula and the basal ganglia (Sarteret al., 2006, Zaborszkyet al., 2018). Several studies reported increased cerebral ACh levels in people suffering from MD, and to a lesser degree also people in remission of MD (Ariaset al., 2021, Charleset al., 1994, Dagyteet al., 2011, Mineur and Picciotto, 2021, Sariciceket al., 2012, Steingardet al., 2000). Evidence suggests that the increased ACh, through inhibitory interneuron stimulation, suppresses pyramidal neurons (Fogacaet al., 2023, Fogacaet al., 2021), leading to reduced extracellular glutamate levels (Kimet al., 2023, Mamelak, 2024) and depression-like symptoms (Wohleb et al., 2017). Drugs that affect the level or effectiveness of available ACh induce depression-like symptoms (Howland, 2009, Mineuret al., 2009, Shytleet al., 2002). Conversely, scopolamine, a selective muscarinic ACh receptor antagonist, has a rapid antidepressant effect in rodents (Fogacaet al., 2023, Wohlebet al., 2017), with treatment potential in humans (Mocko et al., 2023).

The fourth line of research that suggests a broader sensory processing problem in MD consists of resting state functional connectivity studies reporting changed connectivity between the posterior and frontal brain. These include a reduced coupling strength between frontoparietal networks and visual cortices (Chenet al., 2019, Desseilleset al., 2009), increased intrinsic coupling between visual areas (Chen et al., 2019) and altered connectivity between regions in the affective prefrontal cortex and visual and visual-association regions (Samara et al., 2018). These findings suggest an increased autonomy of the visual network in MD patients, characterized by a more economical, efficient internal organization, and less external regulation by the attention network (Chen et al., 2019). Such alterations are consistent with the assumed modulatory influence of ACh on information processing (Bentleyet al., 2011, Demeter and Sarter, 2013).

While the evidence mentioned above supports the idea of a more general sensory information processing deficit in MD, it is unclear whether this deficit is a consequence of the depressed state and/or its debilitating effect on motivational, attentional and cognitive capacities, or whether it is characteristic of MD independent of its manifest state. However, if a similar sensory processing deficit could be demonstrated in people who are at risk of MD without manifesting clinical symptoms, that would place this deficit in the category of MD vulnerability markers. In fact, unusual task-related brain activity has been reported in the posterior brain of at family risk persons, i.e. in the occipital, parietal and temporal cortex (Carewet al., 2013, Gotlibet al., 2010, Joormannet al., 2012, Lisieckaet al., 2013, Macoveanuet al., 2016, Mannieet al., 2010, Miskowiaket al., 2015, Nimarkoet al., 2019, Pilhatschet al., 2014, Talatiet al., 2013, Wolfensbergeret al., 2008). So far, only a few fMRI studies have included both people at risk and patients (Amicoet al., 2012, Lisieckaet al., 2013, Nordet al., 2020, Sharpet al., 2014). Instead of looking for common abnormalities, they directly contrasted the two groups, thus emphasizing vulnerability versus state differences. To identify potentially predisposing markers, it is necessary to focus on what both groups have in common. That is what Samara et al. (2018) did in a functional connectivity study of MD. They found that the changes that participants with family risk of MD had in common with participants suffering from MD were not between subregions of the affective prefrontal cortex, but between some of the affective regions and regions of the posterior brain involved in visual processing and attention allocation. Such findings suggest that even before disease manifestation, persons at risk of MD may have aberrant attention-guided processing of everyday events.

The aim of the present study is to investigate whether a sensory processing deficit may be part of the pathophysiology of MD. This could be the case if participants with a family risk of MD share with participants suffering from MD deviations in brain activity associated with the attention-guided processing of external information. To examen this, fMRI data were collected while participants performed an emotional face perception task. Firstly, a whole brain, group-level analysis of the task-related activity should reveal whether these shared differences are mainly in the regions of the dorsal attention and visual systems. Secondly, a ROI-based group analysis will investigate whether observed task activation differences are also present when processing pictures of neutral faces, and therefore are not specific to the emotional expressions of the faces. So, in contrast to most studies, the neutral face condition will not be used here as a baseline to define “emotional” content, because it is part of our hypothesis that MD patients process neutral faces different from control participants (Filkowski and Haas, 2017). Thirdly, resting state fMRI data were acquired to investigate the functional connectivity between sites of altered task-related activation. If the altered activity stems from an underlying modification of the attentional modulation of sensory processing, this should be reflected in the local inter-regional communication between the affected regions, even during a state of rest, when no task is executed. Lastly, to investigate ACh involvement in the hypothesized information processing deficits, an additional functional connectivity analysis will focus on connectivity difference, again shared by family members and patients, between attentional and visual areas and seeds in the MBF, which hosts the attention-modulating cholinergic neurons.

## 2 Methods

### 2.1 Participants

Task-related resting state fMRI data were collected from 30 participants with a family risk for MD (family history group or FH), 28 participants with current MD (cutoff BDI-II > 20; MD group) and 28 healthy controls matched for age and gender to the first two groups (HC group). All groups underwent one MRI scanning session and behavioral testing. The same participants were all also included in the larger functional connectivity study published elsewhere (Samara et al., 2018). FH participants were selected based on the following criteria: 1) they had a first-degree relative with a current or lifetime MD diagnosis; 2) they had never suffered an MD episode themselves; 3) they were free of any other current or lifetime psychiatric diagnoses. MD participants were included when currently meeting DSM-IV-TR criteria for MD and excluded if they were diagnosed with Bipolar I or II, substance dependence or were taking benzodiazepines. HC participants were excluded in the presence of any current or lifetime axis I diagnosis and immediate or extended family history of any psychiatric disease. The study was conducted according to the principles of the Declaration of Helsinki 2008 and was approved by the institutional medical ethical committee. After the study procedures were fully explained, participants provided informed consent. Recruitment and screening procedures for all participants are described in Supplement, section 1.1 and medication histories of MD patients in Supplement, Table S1.

During the testing session and prior to scanning, participants completed the following self-report scales: Beck Depression Inventory-II (BDI-II) (Beck et al., 1996), Brief Symptom Inventory (BSI) (Derogatis and Melisaratos, 1983), and the Quick Inventory of Depressive Symptomatology Self-Report (QIDS-SR) (Rush et al., 2003). Participants were also assessed with a computerized version of Raven’s Standard Progressive Matrices, a 60-item test of non-verbal IQ (Raven et al., 1998).

### 2.2 Affective faces task

The participants viewed color pictures of happy, neutral, or sad faces during fMRI scanning, and categorized them according to the gender of the face, with a right hand key press. Their reaction time and response accuracy were recorded. All participants obtained an accuracy of more than 95 %. The face stimuli were selected from the Nimstim package (https://danlab.psychology.columbia.edu/content/nimstim-set-facial-expressions) based on the percentage correct identification of the displayed emotion (sad, happy and neutral) (all faces > 80 %) by two independent samples (Tottenham et al. (2009), and our own pilot data). Affective faces were presented in 16 blocks of either neutral and sad or neutral and happy faces (8 emotional and 4 neutral faces per block), alternated with 16 rest blocks (∼32 secs) during which participants fixated on a cross in the middle of the screen. The pictures were presented in random order at the center of the computer screen against a gray background for 2 s, with an average inter-stimulus interval of 2.5 secs that randomly varied in 1 sec steps. This timing of picture succession was chosen to allow for an event related analysis (Dale, 1999, Ollingeret al., 2001), in which the BOLD response induced by each of the three categories of pictures could be estimated independently (see section 2.4.2, below).

### 2.3 Image acquisition & processing of functional imaging data

Scanning was conducted on a Siemens MAGNETOM Allegra 3 T MRI scanner with a 1-channel head coil. The echo-planar imaging (EPI) sequence was optimized to reduce susceptibility artifacts in the orbital, ventromedial and temporal cortex, by using a low echo time (25 ms), a smaller voxel size (2 × 2 × 3.24 mm for task-related and 2 × 2 × 3 mm for resting state scans), a slice orientation parallel to the orbital surface (∼30° angle), and applying field map distortion correction. For the task-related scan 680 T2*-weighted gradient EPI images were acquired with a repetition time of 2.0 s. For the two resting state scans 153 T2*-weighted gradient EPI images were acquired (except for 6 participants with 203 images), with a repetition time of 2.5 s. For each functional scan, a gradient echo image with the same grid and slice orientation generated a field map for distortion correction. Lastly, to allow spatial localization of results, a high-resolution T1-weighted image was acquired with voxel size 1 × 1 × 1 mm. More details on the acquisition parameters are available in Supplement Section 2.1.

The MRI data were pre-processed using SPM12 software (Welcome Trust Center for Neuroimaging, London, UK). This included slice time correction, spatial correction using the field map, realignment, co-registration with the anatomical scan, normalization to the Montreal Neurological Institute (MNI) template (ICBM-152), reslicing to 2 mm isotropic voxels, and smoothing with a 6 mm full width at half maximum Gaussian kernel. The T1-weighted images were segmented into grey matter, white matter, and cerebrospinal fluid tissue maps, which were later used in the analyses. All preprocessing steps used the default SPM12 parameter settings.

The following steps were taken to minimize the influence of head motion during scanning: 1) participants were excluded if in any scan the first-to-last image displacement exceeded 3.0 mm; 2) the volume-to-volume realignment parameters were used as regressors of no interest in the statistical analyses of the data; 3) volumes with a volume-to-volume displacement exceeding 1/10th of the slice thickness were omitted from the analysis; 4) groups were matched on the average absolute volume-to-volume spatial displacement variance; 5) the average relative displacement of each participant was added as a covariate in the 2nd level random effects analyses. A detailed description of these steps is available in Supplement Section 2.3.

### 2.4 Statistical analyses

#### 2.4.1 Behavioral data

Behavioral data were analyzed to test whether the groups differed in accuracy and response speed when identifying gender in the face perception task. These dependent variables were analyzed by fitting a general linear model to the data with group (HC, FH and MD) as a between subject factor, emotional expression of the faces (happy, neutral, sad) as a within subject factor, and age, gender, and Raven nonverbal intelligence as covariates. The Huyn-Feldt correction was applied to correct for violations of the sphericity assumption (see Supplement Section 1.2).

#### 2.4.2 fMRI data, individual level

The task-related functional data of each participant were analyzed with a voxel-wise general linear model (GLM) in SPM12. A model of the signal change induced by the events of interest were created for each participant by convolving a vector of trial onsets for each event type (sad, neutral and happy face stimuli) with a canonical hemodynamic response function and its temporal and dispersion derivatives. The model further included the head motion parameters computed during image realignment and the averaged signal from white matter and CSF. Lastly, stimulus events whose signal time course coincided with excessive volume-to-volume head motion where modelled as separate events of no interest (see Supplement Section 2.3 for details on head motion corrections). The estimated beta-weight maps for each of the three face categories (sad, neutral, and happy) were expressed as a percent signal change relative to the voxel-specific mean signal (Gläscher (2009); https://marsbar-toolbox.github.io/). Therefore, for each participant three percent BOLD signal change maps were created and used as data for the group level analyses described below.

The two resting state data per participant were cleaned by regressing out the variance associated with linear trends, the six volume realignment parameters, the average signal from white matter and CSF, and the session-specific mean. The residual data were 0.01–0.1 Hz band-pass filtered. Next, the volumes with excessive volume-to-volume head motion were removed, together with one volume before and two following the contaminated volume. The two scans per participant were concatenated into one time series data. Next, for each participant a seed-by-seed correlation matrix was created by computing the Pearson correlation between the averaged voxel time courses of a set of seeds. The seeds were created from the voxel clusters that resulted from the vulnerability analysis results described below and from probability masks for the magnocellular basal forebrain nuclei (see below, section 2.4.4).

#### 2.4.3 Task-related fMRI data, group level

MD vulnerability at the brain level was defined as regions whose activation or deactivation during face processing did not differ between the FH and the MD participants, while it was significantly different in the comparison of both these groups with the HC group. To this end, first, the first-level percent signal change maps were entered into a second-level random-effects GLM analysis with the valence of the face stimuli as a repeated measures factor with three levels (percent signal change induced by happy faces, neutral faces, and sad faces) and group as between-subjects factor, also with three levels (HC, FH, MD). Participant’s age, gender, Raven IQ score, and overall head motion (volume-to volume head position variation in the z-dimension) were included in the model as covariates of no interest. Moreover, to control for signal variation related to severity of disease status, depression-related measures were added as regressors in a group-specific manner: BDI-II and PSDI scores in each group, and antidepressant medication and comorbidities in the MD group. This group-specific control was required, because on the one hand, comorbidity and medication status are only applicable to the MD group, and on the other hand, BDI-II and PSDI scores strongly differ between the FH and MD group because of the way the experimental groups were created (see Supplement Section 2.4 for more details and further justification).

In a second step, a conjunction map was constructed from the *F* contrasts between 1) the family history and healthy participants, and 2) MD patients and the healthy participants. In these contrasts, the valence-conditions of the face stimuli were pooled together per group. Only voxels significant at the False Discovery Rate (FDR) (Genoveseet al., 2002, Nichols, 2012) significance level of 0.05 in both *F* contrasts were included in the conjunction map. Finally, all voxels that differed significantly (FDR corrected) between the FH and MD groups were removed from the conjunction map. Based on the inherent smoothness of the data, the cluster size expected based on the Gaussian Field Theory at the FDR corrected significance level was 5.9 voxels for the HC vs FH contrast and 6.5 for the HC vs MD contrast. The number of clusters expected was 1.8. We had 64 clusters in the conjunction map. However, we will only consider here the 29 clusters with 6 or more voxels—i.e., a voxel size equal to or larger than the expected cluster size. These will be referred to as “vulnerability clusters”. For a list of all 64 clusters, see Supplement Table S2.

A potential emotional bias in the brain response of the FH and the MD group compared to the HC group was investigated at the level of individual vulnerability clusters, with a region of interest-based group analysis. For this analysis, the average percent signal change was computed for each cluster and each emotional face category in each participant. These were used as data in a mixed effects model, that included emotional valence (happy, neutral, sad) of the stimuli as within subject factors, the experimental groups (HC, FH and MD) as between subject factor, and the same covariates also used in the whole brain analysis. Emotional bias in the brain response of a cluster would result in a significant interaction between the emotional content of the faces (sad, neutral and happy) and the experimental groups (HC, FH, and MD). The cumulative binomial coefficient was used to control for the cumulating chance of observing false positive results when repeating the test for each cluster at a significance level α = 0.05. See Supplement Section 2.4 for a more detailed description of the mixed effects ANOVA model used.

#### 2.4.4 Functional connectivity data, group level

The resting state functional connectivity between a subset of the vulnerability clusters derived from the group analysis of the face task was investigated with a seed-to-seed correlation approach. For this purpose, the activation clusters were grouped into seven regions of interest (as described in Supplement table S3), each with a left and right hemisphere homologue. A seed-by-seed correlation matrix was computed for each participant. After Fisher-Z transformation, for each seed-pair (i.e., each unique cell in the matrices) a 3 factor ANOVA was performed on the correlation values, with group as a between subject factor (2 levels: either FH versus HC or MD versus HC), and the two seeds each with a left and a right hemisphere homologue as two within subject factors (2x2 levels, e.g., Seed1-left to Seed2-left, Seed1-left to Seed2-right, etc.). Because we are looking for connectivity differences between experimental groups, we will present only the main effect of group and ignore interaction effects between group and brain side of the seeds. To reduce the amount of variance not accounted for by the model, the same covariates as described for the task-related GLM analysis were also included in the current ANOVA model. The seven seed regions involved in the analysis necessitated (7^2^ –7)/2 = 21 tests per pairwise group comparison, each executed at the uncorrected significance level α = 0.05. To guard against the cumulating chance of finding false positives, the cumulative binomial test was used to assess the likelihood of observing an equal or higher number of positive tests under the null-hypothesis. A more detailed description of the analyses and procedures can be found in Supplement Section 2.5.

Seed definitions for the cholinergic cell groups in the magnocellular basal forebrain (MBF) were derived from the probabilistic anatomical map, available in the SPM Anatomy Toolbox (Eickhoff et al., 2007). The MBF maps are based on microscopic delineations of 10 postmortem human brains (Zaborszky et al., 2008). The probabilistic delineations for the ch1-3 and ch4 + cell groups largely correspond to the two functional subdivisions that were recently demonstrated in the basal forebrain (Fritz et al., 2019). The hemisphere-specific probabilistic maps for each cell group were thresholded at a probability of 0.6 and resampled to the voxel space of the normalized resting state images. For each of the resulting left and right MBF seed regions an average time course was computed and correlated with the average time courses of each other and the seven bilateral vulnerability seeds. Each cell of this correlation matrix was again subjected to an ANOVA with the same model structure as described in the previous paragraph. By crossing the two MBF seeds with each of the seven vulnerability seed regions, 15 unique tests were required, each executed at the uncorrected significance level α = 0.05. Again, the cumulative binomial test was used to assess the likelihood of observing an equal or higher number of positive tests under the null-hypothesis.

## 3 Results

### 3.1 Demographic and clinical characteristics

The characteristics of the three participant groups are summarized in Table 1. The groups were matched for age, gender and IQ (all *p*’s > 0.2). Overall, participants were in their mid-thirties and ± 70 % were females. The FH group did not differ significantly from the HC group in scores of depressive and general psychiatric symptomatology (see Table 1). MD participants at the time of the scan had on average BDI-II and QIDS-SR scores of 32.3 and 16.1 respectively. Thus, average depression severity of the participants in the MD group was in the severe range.

**Table 1:** Participant characteristics and clinical measures.

	MD		FH		HC			
	Mean	SD	Mean	SD	Mean	SD	*F* (2,83)	*p*
BDI-II	32.32	7.26	4.53	3.88	2.68	3.30	300.34	0.000^a^
BSI GSI	1.63	0.53	0.23	0.22	0.15	0.18	164.66	0.000^a^
BSI PST	38.29	8.11	10.03	8.29	6.79	7.27	135.30	0.000^a^
BSI PSDI	2.22	0.43	1.09	0.27	0.88	0.44	99.52	0.000^a^
QIDS	16.14	3.16	3.03	2.22	2.25	2.01	274.00	0.000^a^
Age (yrs.)	39.21	12.29	33.23	14.28	35.54	16.63	1.25	0.293
Female %	68 %	−--	73 %	−--	71 %	−--	χ^2^(2) = 0.22	0.898
Raven IQ^b^	45.93	7.13	48.63	6.16	45.68	7.15	1.69	0.190

Note. MD, major depression group; FH, familial history of depression group; HC, healthy control group; BDI, Beck Depression Inventory [68]; BSI, Brief Symptom Inventory [69]; GSI, global severity index; PST, positive symptom total; PSDI, positive symptom distress index; QIDS, Quick Inventory of Depressive Symptomatology Self-Report (Rush et al., 2003). ^a^ The MD group differs significantly from relatives and controls. ^b^ T-scores with mean 50 and standard deviation 10.

### 3.2 Behavioral data

The three groups did not differ significantly in accuracy (*F* (2, 252) = 1.08, *p* = 0.343) or response time (*F* (2, 252) = 1.55, *p* = 0.214) of identifying the gender of the presented faces. There was also no interaction between the group to which a participant belonged and the emotional content of the faces in the accuracy (*F* (3.60, 453.94) = 0.15, *p* = 0.951) or the response time (*F* (3.84, 483.61) = 0.235, *p* = 0.913).

In contrast, the emotion expressed by the faces did in general have a significant effect on the speed of responding (*F* (1.92, 483.61) = 7.77, *p* < 0.001) across groups, but not on the accuracy (*F* (1.80, 453.94) = 2.63, *p* = 0.079). Therefore, while this result shows the validity and effectivity of manipulating the emotional content of the faces, the behavioral data do not provide support for an emotional bias in the FH participants or the MD patients.

### 3.3 BOLD markers of MD vulnerability during face processing

**Fig. 1:**
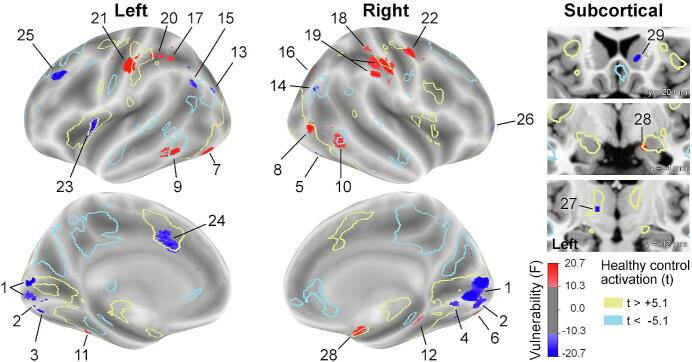
Vulnerability clusters overlaid on an inflated brain template. Clusters of increased activity (red; HC < FH, MD and FH = MD) and decreased activity (blue; HC > FH, MD and FH = MD) are in filled color. The outlines of voxel clusters represent activation (yellow) and deactivation (light blue) patterns induced by the task execution in the HC group. Cluster ID numbers refer to entries to Table 2. (For interpretation of the references to colour in this figure legend, the reader is referred to the web version of this article.)

The whole-brain analysis of task-related activity revealed 29 clusters of 6 or more voxels, in which the BOLD signal differed significantly between the HC group and both the MD and the FH group, while it did not differ between the latter two groups. The clusters are detailed in Table 2, while Fig. 1 shows their location in the brain relative to the areas of task-induced (de)activation, and the direction of their BOLD change.

**Table 2:** List of all clusters of voxels with different activity in the healthy control (HC) compared to the family history of depression (FH) and major depression (MD) participants, while activity in the latter two groups did not differ.

MD vulnerability cluster description							Functional characteristics									
											Percent signal change^d^					
			Center of mass (mm)				Relation		Direction		HC		FH		MD	
ID	Location^a^	Side	x	y	z	N vxls	to task cortex^b^		of change^c^		M	SD	M	SD	M	SD
Lower visual areas																
1	Calcarine sulcus	Bilateral	8.0	−80.3	5.0	185	task pos.			neg.	0.62	0.38	0.34	0.26	0.30	0.32
2	Lingual gyrus	Bilateral	4.0	−90.8	−8.9	16	task pos.			neg.	1.43	0.64	1.09	0.59	1.08	0.56
3	Lingual gyrus	Left	−6.9	−81.3	−16.1	25	task pos.			neg.	0.88	0.55	0.56	0.52	0.55	0.39
4	Lingual gyrus	Right	18.4	−77.1	−21.9	15	task pos.			neg.	0.26	0.41	0.10	0.22	0.11	0.20
5	Lingual gyrus anterior	Right	4.7	−65.0	3.0	6	task pos.			neg.	0.41	0.50	0.21	0.37	0.19	0.29
Ventral visual																
6	Collateral sulcus	Right	24.7	−89.5	−10.5	12	task pos.		pos.		0.42	0.42	0.72	0.44	0.66	0.39
7	Fusiform gyrus posterior	Left	−39.4	−77.0	−17.1	14	task pos.		pos.		0.77	0.50	1.23	0.72	1.23	0.57
8	Lateral occipital cortex	Right	53.3	−72.6	0.0	60	task pos.		pos.		0.26	0.26	0.48	0.32	0.51	0.35
9	Inferior temporal sulcus	Left	−61.3	−52.1	−13.3	16		de-activ.	pos.		−0.24	0.22	−0.12	0.19	−0.09	0.25
10	Inferior temporal sulcus	Right	56.2	−52.0	−5.3	9		de-activ.	pos.		−0.12	0.15	−0.02	0.17	0.00	0.18
11	Fusiform gyrus	Left	−25.3	−45.3	−19.0	6		de-activ.	pos.		0.00	0.21	0.10	0.20	0.11	0.26
12	Fusiform gyrus anterior	Right	16.7	−34.7	–22.9	14		de-activ.	pos.		−0.05	0.16	0.06	0.14	0.05	0.17
Dorsal Visual																
13	ventral Intraparietal sulcus	Left	–32.9	−89.1	26.3	7	task pos.			neg.	−0.02	0.34	−0.20	0.29	−0.15	0.36
14	ventral Intraparietal sulcus	Right	40.0	−78.0	33.0	6		de-activ.		neg.	−0.18	0.20	−0.34	0.41	−0.28	0.23
15	Intraparietal sulcus	Left	−40.3	−63.1	36.8	13		de-activ.		neg.	0.00	0.19	−0.10	0.19	−0.11	0.16
16	Intraparietal sulcus	Right	35.3	−61.3	29.0	12	−	−	pos.		−0.05	0.11	0.04	0.16	0.04	0.14
Parietal and sensorimotor																
17	Intraparietal sulcus	Left	−43.3	−52.8	51.3	8	task pos.		pos.		−0.06	0.22	0.07	0.21	0.09	0.24
18	Intraparietal sulcus	Right	44.8	−27.2	43.2	10	task pos.		pos.		0.15	0.11	0.21	0.14	0.23	0.14
19	Postcentral/intraparietal sulcus	Right	57.7	−21.4	46.8	101	task pos.		pos.		0.10	0.16	0.26	0.22	0.27	0.20
20	Intraparietal sulcus	Left	−34.8	−9.8	14.0	19	task pos.		pos.		0.09	0.09	0.16	0.16	0.17	0.14
21	Postcentral/intraparietal sulcus	Left	−53.0	−18.4	43.5	44	task pos.		pos.		0.04	0.22	0.17	0.14	0.19	0.18
22	Superior frontal/precentral sulcus	Right	41.5	−5.4	53.3	22	task pos.		pos.		0.05	0.18	0.14	0.17	0.16	0.17
Prefrontal (and insular)																
23	Insular cortex	Left	−34.0	4.8	8.2	10	task pos.			neg.	0.02	0.12	−0.04	0.13	−0.04	0.13
24	Cingulate sulcus (aCMA)	Left	−6.7	13.1	36.0	11	task pos.			neg.	0.17	0.13	0.11	0.12	0.12	0.12
25	Middle frontal gyrus	Left	−34.1	33.1	41.1	16		de-activ.		neg.	−0.02	0.16	−0.12	0.19	−0.13	0.16
26	Frontal Pole	Right	24.7	64.6	0.8	20	−	−		neg.	0.31	0.24	0.13	0.14	0.22	0.18
Subcortical																
27	Ventral thalamus	Left	−14.0	−12.0	1.5	6	task pos.			neg.	0.08	0.10	0.02	0.09	0.02	0.10
28	Amygdala	Right	17.5	−4.5	−15.5	11	task pos.		pos.		0.15	0.26	0.30	0.41	0.30	0.37
29	Ventral striatum	Right	13.9	19.3	0.1	14	−	−		neg.	0.03	0.13	−0.08	0.12	−0.05	0.12

Note. Clusters are roughly listen in the order of their anterior-dorsal position (Y-dimension), and group according to functional properties.

Abbreviations: vxls = voxels; aCMA = anterior cingulate motor area.

a For specific anatomical locations of visual areas we followed (Stiers et al., 2006).

b Location of vulnerability cluster in or near task-modulated cortex, i.e., either task-activated (“task pos.”) zones or task-de-activated (“de-activ.”) zones in the HC group.

c Activity level of FH and MD groups is either higher (pos.) or lower (neg.) than in the HC group.

d Percent signal change across all trials (i.e., regardless of affective expression of the face stimuli), in 10% most responsive voxels in each cluster, or all voxels for clusters with less than 10 voxels.

Inspection of Fig. 1 makes clear that most of the clusters are located in the posterior, receptive brain, with only three clusters in the prefrontal cortex (clusters 24–26 in Table 2). This suggests that the bulk of indicators for depression risk in our data consists of changes in the reception of external information. Most of the clusters (19, or 65.5 %) are located in or adjacent to task-related activation zones, and another 7 (24.1 %) are located in or near areas that are systematically de-activated during task performance. Therefore, 89.6 % of the clusters are directly or indirectly related to the commitment inherent in executing the task. Of these, 4/5th separates into four groups associated with attentional processing of visual information. One group is positioned in the lower visual cortex, including the calcarine sulcus and lingual gyrus bilaterally (Stiers et al., 2006). A second group comprises of clusters in the lateral and ventral association areas of the “ventral” visual stream (Stiers et al., 2006), involving bilateral fusiform gyrus and inferior temporal sulcus, as well as the right hemisphere collateral sulcus and lateral occipital cortex. The third group occupies the dorsal visual cortex situated around the ventral intraparietal sulcus (Stiers et al., 2006). Lastly, six clusters are located in cortical areas associated with the dorsal attention system (Corbetta and Shulman, 2002, Stierset al., 2006), i.e., the cortical zone where precentral sulcus merges with the superior frontal sulcus and the post-central sulcus with the intraparietal sulcus. The remaining 1/5th of clusters related to task execution is located in the left hemisphere of the frontal brain, namely in the middle frontal gyrus, the cingulate sulcus and the insula, or subcortically, in the left thalamus or the right amygdala. The three clusters not associated with task-modulated cortex are located in the right frontal pole, the right ventral striatum, and in the right intraparietal sulcus (clusters 26, 29 and 16, respectively, in Table 2).

When the direction of change in the task-induced BOLD response is taken into account, the following spatial pattern becomes apparent. On the one hand, clusters of increased BOLD activity in the FH and MD participants tend to be located in the cortical region associated with the dorsal attention system, and with lateral and ventral association areas of the “ventral” visual stream. On the other hand, clusters where MD risk is associated with reduced task-induced activity are located in the lower tier visual areas and in the ventral aspect of the dorsal visual stream.

### 3.4 Emotional valence

To assess whether the deviant brain activation in the 29 clusters was specifically driven by the emotional content of the face stimuli, ROI-based group analyses of the average percent signal chance in each MD vulnerability cluster were performed. In only 2 of the 29 clusters the interaction effect between emotional content and groups was significant, namely in the right lingual gyrus (cluster 4 in Table 2: *F* (4.0, 142.0) = 3.97, *p* = 0.004), and in the left fusiform gyrus (cluster 7 in Table 2: *F* (3.91, 138.70) = 4.42, p = 0.002). The probability of 2 or more false positive tests out of 29 is p = 0.429. Therefore, we have no ground for accepting these two tests as true positive result.

### 3.5 Functional connectivity

**Fig. 2:**
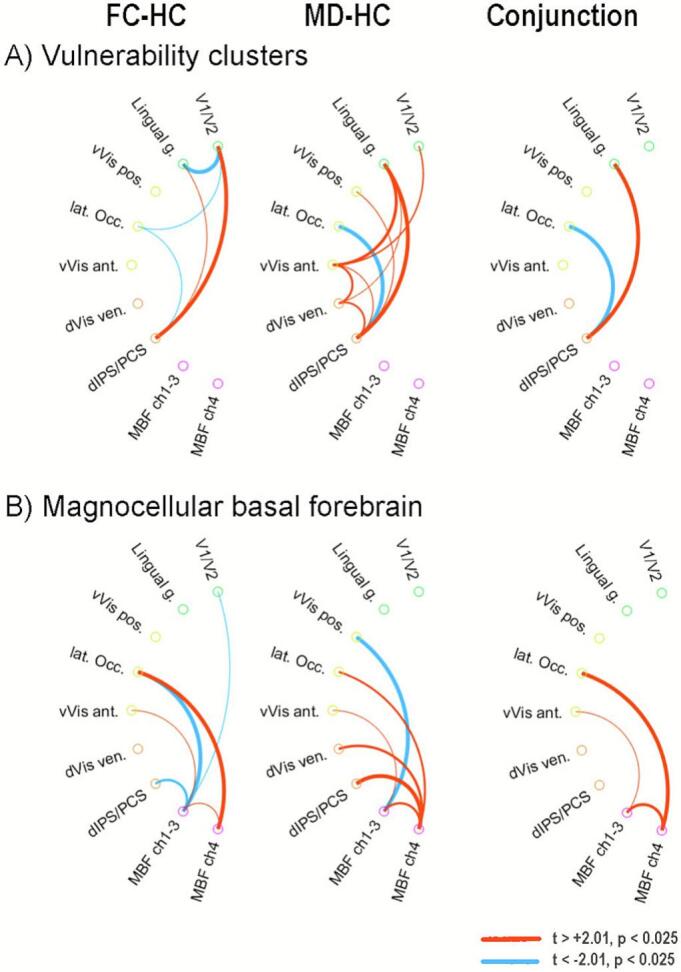
Functional interconnectivity changes between MD vulnerability seeds (A), and connectivity changes of vulnerability seeds with the two major magnocellular basal forebrain nuclei (B). Data are presented for changes observed in the FH participants versus HC participants, in MD participants versus HC participants, and the conjunction map (FH, MD ∼= HC). Shown are connection differences significant at 2-tailed alpha = 0.05. Line thickness is proportional to t-statistic, or the averaged t-value for the conjunction graph. vVis, ventral visual; dVis ven, dorsal visual–ventral section; dIPS, dorsal intraparietal sulcus; PCS, postcentral sulcus; MBF, magnocellular basal forebrain; ch1-3 and ch4, cholinergic cell groups 1 to 3, and 4, respectively.

Fig. 2A left summarizes the resting state functional connectivity changes between the seven vulnerability seeds in the FH versus HC comparison. Five of the 21 tests were significant at α = 0.05 (cumulative binomial p < 0.003). The changes consisted of increased low frequency coupling between the dorsal parietal seeds and the lower-tier visual areas, together with reduced coupling amongst these lower visual areas, suggesting increased regulation originating from the dorsal attention system, leading to reduced processing of visual information, particularly in the more peripheral visual field. At the same time, these lower visual seeds and the dorsal parietal seeds showed reduced coupling with the lateral occipital cortex.

Functional connectivity differences between the vulnerability seeds in the MD versus HC comparison are summarized in Fig. 2A middle. Of the 21 pairwise tests, 9 were significant at α = 0.05 (cumulative binomial p < 0.001). The differences partially overlap with those observed in the FH versus HC comparison, namely increased coupling of the dorsal parietal seeds with the lingual gyrus, together with reduced coupling with the lateral occipital cortex (Fig. 2A right). However, the MD group did not show increased coupling of the dorsal parietal seeds with V1/V2. Instead, functional coupling was increased with higher visual association areas. This suggests that in the state of depression, in contrast to MD risk, attentional regulation is directed at categorization and identification of visual input, rather than the filtering of lower-level incoming information observed in FH group.

To investigate the involvement of ACh modulation mechanisms in the brain changes observed, the functional connectivity of the 7 vulnerability seeds with the two MBF seeds were investigated (Fig. 2B). The number of significant tests was 6 out of 15, for both the MD-HC and the FC-HC comparison (cumulative binomial p < 0.001). The deviations from the HC group shared by the FH and MD group are depicted in Fig. 2B right, and consisted of an increased coupling between the ch4 cell group and the lateral occipital complex and between the ch1-3 cell groups and anterior visual association areas. Moreover, there was a stronger similarity in the time courses of the two MBF seeds in both groups. In the FH group, additionally, the ch1-3 cell group showed reduced functional connectivity with vulnerability regions at the lower (V1/V2), intermediate ventral (LOC) and highest dorsal (dIPS/PCS) levels of visual processing (Fig. 2B left). In contrast, in participants with MD, the ch4 cell group showed stronger coupling with several regions at the intermediate and higher level of dorsal and ventral visual processing, while the medial cell groups (ch1-3) had reduced coupling with the posterior ventral visual area (Fig. 2B middle).

## 4 Discussion

This study investigated whether alterations in sensory processing observed in people with MD reflect the mood changes associated with the manifestation of the disease, or alternatively whether these alteration characterize the disease regardless of its onset state. To this end, brain activity was compared of participants with MD, participants without MD but at risk because of an MD family member, and healthy control participants, while they engaged in a gender identification task using pictures of faces with a sad, neutral, or happy expression. Resting state functional connectivity data were also collected to look at possible altered functional couplings between involved brain regions. The underlying idea was that brain deviations characteristic of the disease (instead of just being the consequence of disease manifestation) should be discernable in people who are at increased risk for MD without having suffered an MD episode. The existing literature provided hypotheses about what shared brain activity alterations could be expected, given a general sensory processing deficit. In line with these expectations, the whole-brain task-related analysis revealed clusters of altered activity mostly in posterior brain regions involved in attentional modulation and processing of the face stimuli. A ROI-based analysis of these clusters showed that the alterations were equally present in the neutral faces, and therefore not specific to the emotional task conditions. Functional connectivity analysis established that the altered sensory processing was secured in the functional couplings between attention related and visual areas even when participants were not executing the task. Finally, in agreement with the elevated cortical ACh-levels observed in people with MD, several of the clusters of changed task activation had altered functional connectivity with the MBF, the brain structure from which the modulatory ACh signals originates. These findings and their implications will be discussed in more detail below.

### 4.1 Emotional processing deficit

The emotional face paradigm was chosen to tap into the affective dimension of MD symptomatology. However, our results provide no support for a (negative) emotional bias in MD patients or family members of MD patients, not in gender identification performance, nor in brain responses to the emotional content of the stimuli. We did however find evidence for a different “emotional” brain response, evident in all stimulus categories, that was shared by the participants suffering from or at risk of MD. This consisted of an increased activation in the right amygdala and reduced activity in the right ventral striatum and the left anterior cingulate motor area implicated in action-outcome updating (Monosovet al., 2020, Rushworthet al., 2004). It could be the case, therefore, that compared to the HC group, the brains of FH and MD participants reacted as if the face stimuli in generally were more affectively salient and less rewarding or motivating to perform the task.

Despite these alterations in the response of affect/motivation-related brain structures, no task-related activation changes were observed in the affective regions of the frontal lobe, i.e., the orbital and anterior medial prefrontal cortex. This negative result confirms the finding by Samara et al. (2018), in the same sample, that there were no changes in functional connectivity between the subregions of this affective prefrontal cortex. Increased activity in the pre- and subgenual parts of the affective prefrontal cortex is occasionally reported in studies of emotional face perception (Groenewoldet al., 2013, Jaworskaet al., 2015) and of broader emotional/cognitive tasks (Grahamet al., 2013, Pizzagalli, 2011). However, these cortical structures are commonly not implicated in studies of emotional face perception in participants at risk of MD (Amicoet al., 2012, Lisieckaet al., 2012, Mannieet al., 2011, Monket al., 2008, Pilhatschet al., 2014, Simseket al., 2017, Swartzet al., 2015, van der Veenet al., 2007), and were observed by Watters et al. (Watters et al., 2018) only under subliminal presentation conditions, when emotion regulation cannot occur. It seems, therefore, that changed activation in the affective prefrontal cortex is not a vulnerability feature, but a consequence of the acute MD state. In support of this interpretation, in remitted MD patients hyper-activation of ventromedial prefrontal cortex was found to be predictive of relapse risk (Farb et al., 2011), and abnormal activity in the pre- and subgenual cingulate cortex has been reported to improve with treatment (Ma, 2015, Price and Drevets, 2012) (albeit not always (Schoning et al., 2009)).

### 4.2 Sensory processing deficit

Most of the activation changes fitting our operationalization of vulnerability were found in the posterior brain, and they were linked to areas of task-related (de-)activation – i.e., the dorsal attention system, the ventral and lateral visual object identification system and the lower tier representations of the peripheral visual fields. This finding again corroborates the conclusion arrived at by Samara et al. (2018), from functional connectivity analysis of the same sample, that vulnerability for depression is manifested mainly as a change in the neural processing of environmental events. The fact that we observed these changes also during the categorization of neutral faces further confirms this conclusion. These results support the growing group of publications that show a more general information processing deficit in MDD. This deficit is not only manifested in tasks with an explicit emotional content, but also in purely cognitive tasks focusing on working memory (Mannieet al., 2010, Nordet al., 2020, Watterset al., 2019) or executive response inhibition (Hoffmannet al., 2019, Watterset al., 2019), in perceptual tasks probing visuospatial attention (Cavezianet al., 2007, Pardoet al., 2006) or coherent motion detection (Nortonet al., 2016, Songet al., 2021), and even psychophysical measures of binocular rivalry (Jiaet al., 2020, Jiaet al., 2015) and contrast sensitivity (Bublet al., 2009, Famet al., 2013).

In our study, we did not find behavioral evidence of impairment in the MD group or the FH group, because the cognitive and perceptual demands of our task were very low. However, even under these low demanding conditions, the task-related activation patterns showed alterations in neural sensitivities, alterations that were also detectable in the task-independent low frequency couplings between some of the brain structures involved. It is an important finding that these neural processing deficits were already identifiable in people at risk for the disorder, before the first episode of mood dysfunction.

### 4.3 ACh involvement in the deficit

The contrast between dorsal attention and lower visual areas in vulnerability related activity changes is reminiscent of the modulatory influence that acetylcholine (ACh) has on visual information processing (Bentleyet al., 2011, Demeter and Sarter, 2013, Sarteret al., 2006, Sarteret al., 2014). The normal functionality of ACh is to free lower level sensory processing networks from top-down influences in a precise and task-specific manner. ACh enhances the encoding and processing capacity of local circuitry by desynchronizing and decorrelating neuronal activity (Zaborszky et al., 2018), leading to increased signal-to-noise contrast in the cortex (Minces et al., 2017). These effects are thought to promote unbiased bottom-up information processing, increasing the detectability of stimuli. In this respect, we observed an opposite pattern of activity changes for lingual gyrus (LG) and the lateral occipital complex (LOC): in the LG voxels increased coupling with the dorsal intraparietal/postcentral vulnerability voxels (dIPS/PCS) co-occurred with reduced activity, while the LOC voxels show reduced coupling with dIPS/PCS voxels and increased activity. The increased coupling between dIPS/PCS and lingual gyrus (and V1/V2 in at risk participants) may reflect an increased interference of attentional processes that selectively suppresses (irrelevant) visual processing, leading to decreased activation in these lower visual areas, particularly in the peripheral visual field representations. This is consistent with the reported reduced activity in V4 of MD patients during a visual attention task (Desseilles et al., 2009), in combination with an increased effective influence of IPS over V4 and over V1 to V4 feedforward communication (Desseilles et al., 2011). In contrast, the reduced coupling of dIPS/PCS with the LOC is consistent with an increased independence of LOC (and higher visual association cortex) from top-down attentional interference, leading to higher task-related activity. In line with this, Le et al. (2017) found that the visual association cortex of participants with MD showed reduced working memory updating activity and persistent processing of no longer relevant visual stimuli. Because the elimination from working memory of obsolete items is a top-down process, this finding confirms that higher visual processes operate more independent from prefrontal and parietal feedback. Also in line with this conclusion, Chen et al. (2019) found in a large sample functional connectivity study, that MD is associated with increased internal clustering within the dorsal and the ventral visual networks, together with decreased internetwork connectivity with the dorsal attention network.

These indications of increased autonomy in visual association cortex could be related to the increased ACh availability observed in people with MD (Charleset al., 1994, Sariciceket al., 2012, Steingardet al., 2000). This explanation is supported by our finding of increased functional coupling of key vulnerability regions, in the FH and MD groups, with the two MBF cell groups (Fritz et al., 2019). We found a significantly increased coupling between activity fluctuations in basal nucleus of Meynert (MBF ch4) and LOC. In addition, in both participant groups there was a stronger coupling between MBF ch1-3 and the anterior ventral visual voxels, and between the two MBF seeds. That FH participants shared these deviations with the MD participants suggests a predisposing role of ACh abnormalities in the events that potentially lead to MD.

### 4.4 Limitations and remaining questions

The current findings are novel and exciting but also need replication. One limitation of our study might be a lack of power to detect effects for each emotional valence separately and, consequently, leaving undetected more subtle differences in brain activation related to emotion expression. Furthermore, presenting our neutral faces within blocks of emotional faces (either sad or happy) may have resulted in their processing being contaminated by the affective impact of the embedding negative or positive context. In addition, it remains open how crucial the use of face stimuli was in obtaining our results. Faces are biologically, socially and emotionally highly salient entities. That status may be responsible for triggering the changed responsiveness in the FH and MD participants. On the other hand, the few available studies that did use neutral stimuli in people with acute MD revealed brain activation and connectivity changes that are in line with the results reported here (Chenet al., 2019, Desseilleset al., 2009, Desseilleset al., 2011, Leet al., 2017).

Our study cannot answer the question whether vulnerability characteristics at the neurobiological level translate to behavioral effects, since the task participants were asked to perform was simple gender categorization. A task yielding larger performance variability would allow to investigate the link between the degree of brain activity abnormality and the severity of the performance deficiency. However, such a task would also complicate the interpretation of results by adding factors related to cognitive demands as a plausible explanation for the observed correlation between brain activity and performance. This would muddle the attribution of group differences to disease condition, which were the main focus of this study. Nonetheless, it remains important to establish whether the observed differences in neural processing of face stimuli impact the social and emotional functioning of individuals at risk for MD.

While the present study provides evidence for a sensory processing problem in MD and points to a possible contribution of altered ACh neurotransmission, it remains to be investigated how this sensory problem fits in the pathophysiology of the disease. While manipulating ACh levels can induce or reverse mood-like symptoms (Fogacaet al., 2023, Howland, 2009, Mineuret al., 2009, Shytleet al., 2002, Wohlebet al., 2017), it is not clear how an ACh mediated disturbance of attention-guided sensory processing comes about, or how it can lead to a depressed state. Increased ACh levels can arise due to release or re-uptake problems in the target region, or overstimulation of the cholinergic cells in the MBF source region. In this respect, it is worth noting that we found deviant task-related activity in three of the major telencephalic afferents to the MBF cholinergic cell groups, namely in anterior insula, ventral striatum and amygdala (Sarteret al., 2006, Zaborszkyet al., 2018).

Finally, it is important not to overstate the clinical significance of the findings reported here. Before the observed vulnerability characteristics can qualify as true biological markers of the disease, prolonged and dedicated research is needed, that is directed at establishing their predictive validity and diagnostic specificity. For now, we have established that the abnormalities previously observed in attention-guided sensory processing of patients with MD are not merely a consequence of the diseased state, but are already discernible before the onset of the depressed state.

## CRediT authorship contribution statement

**Peter Stiers:** Writing – review & editing, Writing – original draft, Visualization, Supervision, Software, Project administration, Methodology, Formal analysis, Conceptualization. **Zoe Samara:** Writing – review & editing, Writing – original draft, Project administration, Methodology, Investigation, Formal analysis, Conceptualization. **Kyran J.R. Kuijpers:** Writing – review & editing, Writing – original draft, Formal analysis. **Elisabeth A.T. Evers:** Writing – review & editing, Project administration, Funding acquisition, Conceptualization. **Johannes G. Ramaekers:** Writing – review & editing, Supervision, Project administration, Data curation, Conceptualization.

## Declaration of competing interest

The authors declare that they have no known competing financial interests or personal relationships that could have appeared to influence the work reported in this paper.

## Data Availability

Data will be made available on request.
