# Supplementary material for: Depression vulnerability involves brain activity and connectivity changes consistent with cholinergic deviancy
Source: Neuroimage Clin. 2025 Dec 31;49:103941. doi: 10.1016/j.nicl.2025.103941 (PMC12811235; doi:10.1016/j.nicl.2025.103941)
Supplement: Supplementary Data 1 [file mmc1.docx]

Supplement

Supplement to: Stiers, Samara, Kuijpers, Evers, and Ramaekers, Depression vulnerability involves brain activity and connectivity changes consistent with cholinergic deviancy.

# 1. Participants and behavioral data

## 1.1. Design & Participants

Twenty-eight MD participants currently experiencing a major depressive episode (cutoff BDI-II > 20; mean age 39.2; SD 12.3; 68% female), thirty unaffected first-degree MD relatives (mean age 33.2; SD 14.3; 73% female) and twenty-eight healthy controls (mean age 35.5; SD 16.6; 71% female) participated in the study after providing informed consent. The three groups did not differ significantly in terms of age (F (2, 83) = 1.25; p=.29), gender (χ2 (2) = 0.22; p=.90) or IQ (F (2, 83) = 1.69; p=.19). Nonetheless, to further remove variability associated with these parameters, we included them as covariates in our fMRI analyses.

Participants in all three groups were recruited via advertisements in the local press; MD patients were also recruited from the regional institute for outpatient mental healthcare (RIAGG, Maastricht NL). MD patients recruited from the community were screened for axis I disorders with SCID-I (DSM-IV structured clinical interview) (1) and BDI-II by the experimenter (Clinical Psychologist, MSc.) and trained research assistants. Outpatients of the regional institute for mental healthcare were interviewed for axis I disorders with SCID-I at their intake at the center. All aspects of our screening and experimental procedure were approved by the Medical Ethical Committee of the Academic hospital of Maastricht University and conducted in accordance with the University’s and Committee’s guidelines.

MD patients were included if they met DSM-IV-Text Revision criteria for major depressive disorder as the primary diagnosis and had a BDI-II score of >20 (moderate to severe depression) and excluded if they met criteria for Bipolar I or II, substance dependence or were taking benzodiazepines. MD patients with additional axis I diagnoses (except substance dependence and psychotic disorders) or taking antidepressants were allowed to participate. Table 1 details the diagnostic and medication histories of MD participants. To exclude the possibility that our main results were driven by comorbidity and medication use in part of our MD sample we filtered out from our analyses voxels significant in associated comparisons (for details see section on MRI analyses).

Participants with familial MD were included if they had a first-degree relative with MD, as detailed in a screening questionnaire administered to them. The questionnaire included DSM-IV items for the diagnosis of MD and the differential diagnosis of Bipolar I, II and psychotic features. Participants in this group were excluded if they had been personally diagnosed with any axis I disorder and were assessed with the Symptoms-Checklist 90 (SCL-90) (2) for current psychiatric symptomatology (exclusion cutoff for males SCL-90 > 116, females SCL-90 > 130; above average population norms).

Healthy controls were excluded if they had ever been diagnosed with any axis-I disorder and if they had a first- or second-degree relative with psychiatric history. They were assessed with the SCL-90 for current psychiatric symptomatology and were excluded based on the same cutoffs as the familial MD group. All participants were screened for the following somatic conditions and MRI contra-indications: neurological disorders, epilepsy, severe head injury, claustrophobia, pregnancy or lactation, metal implants, pacemakers and intrauterine contraceptive devices.

## 1.2. Analysis of behavioral data

The performance measures on the face perception task, gender identification accuracy and response time, were analyzed with the IBM SPSS statistics (version 25) software package. For each performance measure separately, a two-way mixed analysis of variance was performed, with experimental group (HC, FH and MD) as a between subject factor and emotional expression of the faces (happy, neutral, sad) as a within subject factor. The age of the participants, their gender and their Raven nonverbal intelligence score were included as covariates of no interest.

Because the analyses include a within-subject factor of valence, it is necessary to deal with the sphericity of the data. The univariate test of SPSS is more powerful than the multivariate test when sphericity is not strongly violated, i.e. when *ε* > .70 (Stevens, 2002). In the analysis of both performance measures the sphericity assumption was violated (Mauchly’s test: *ε* = 0.86 for Accuracy and *ε* = 0.93 for Response Time; in both cases *p* < .001), but was well above 0.70. Therefore, the univariate test was chosen. Furthermore, because *ε* was well above 0.75, the recommended correction for sphericity violations is the Huyn-Feldt correction (Fields, 2018).

# 2. Magnetic Resonance Imaging Acquisition & Preprocessing

## 2.1. Acquisition.

Scanning was conducted on a Siemens MAGNETOM Allegra 3T MRI scanner with a 1-channel head coil. Head motion was constrained by the use of foam padding. For each subject three functional scans were performed: A resting state scan, a task related scan and a second resting state scan. In between non-functional scans were performed: an anatomical scan, a DTI scan and field map scans.

The EPI sequence used for functional imaging was optimized for minimization susceptibility artifacts in orbital, medial frontal and temporal regions that are of interest in emotion processing, by reducing the echo time to 25 ms, choosing smaller voxels and thinner slices (2.0x2.0x3.25 mm), tilting the slice orientation to parallel the orbital plane (~30° angle), and applying field maps to correct local distortion.

For the task related functional scan, 680 T2*- weighted gradient echo planar images (EPI) were acquired using the following parameters: TR, 2000ms; TE, 25ms; flip angle, 90^o^; matrix size, 128 X 96; and FOV, 256mm; distance factor, 20%; number of slices, 33; voxel size, 2.0x2.0x3.24mm. A gradient echo image with the same grid and slice orientation as the functional images (TR, 704ms; TE, 5.11, 7.57 ms; flip angle, 60^o^) was used to generate a field map.

For each resting state functional scan, 153 T2*-weighted gradient EPI images with 41 slices were acquired (except for 6 participants with 203 images available). Imaging parameters for the resting state sequence were as follows: TR, 2500 ms; TE, 25s; flip angle, 90°; matrix size, 128 × 96; and FOV, 256 mm; distance factor, 20%; number of slices 41; voxel size of 2.0 × 2.0 × 3.0 mm. The gradient echo image used to generate the field map had the same grid and slice orientation as the functional images (TR 704 ms; TE 5.11, 7.57 ms; flip angle 60°).

To enable localization of functional data, a high-resolution T1-weighted image was acquired with parameters: TR, 2250 ms; TE, 2.6 ms; flip angle, 9^o^; FOV, 256 mm; matrix size, 256X256; number of slices, 192; voxel size, 1.0x1.0x1.0 mm.

In addition to the 86 participants described above, 9 participants were excluded from the study because the data collection remained incomplete: 4 in the HC group, 1 in the FC group and 4 in the MD group.

## 2.2. Preprocessing.

Preprocessing of fMRI data was performed using SPM 12 software (3) and MATLAB scripts (Mathworks, Natick, MA, USA, version 9.6). The functional data were subjected to the following preprocessing: slice time correction, spatial correction using the field map, realignment, co-registration with the anatomical scan, normalization to the Montreal Neurological Institute (MNI) template, reslicing to 2 mm isotropic voxels and smoothing with a 6 mm full width half maximum (FWHM) Gaussian kernel. The T1-weighted images were segmented into grey matter, white matter and CSF tissue maps which were later used in the analyses.

The resting state scans were further pre-processed by applying a regression analysis to remove the effect of non-neuronal contributions to the BOLD signal from the data. This regression analysis included the following nuisance variables: the six volume realignment parameters, the average time series in white matter and CSF voxels, the session-specific mean, and the intrinsic autocorrelations. The global brain signal or average grey matter signal was not included as a regressor. Finally, the residual data of the multiple regression were Fourier band-pass filtered to remove signal fluctuations beyond the range of 0.01–0.1 Hz.

## 2.3. Head motion management.

During the preprocessing step we calculated various metrics of frame-wise displacement. In order to reduce the bias of head motion in our data we took the following approach:

1) We excluded from further analysis all subjects with translation distance > 3mms over the entire run. Data from three participants (two in the MD group and 1 in the FH group) had to be excluded from further analysis because of this criterion.

2) We estimated the volume-to-volume head motion and identified volumes during which the volume-wise displacement exceeded the following threshold: 1) absolute motion difference in the z direction > 0.4mm; 2) rotation in the x direction > 0.26°, i.e., the angle corresponding to 0.4mm z-displacement of frontopolar voxels, assuming the rotation point in the middle of the brain is 88mm from the anterior end of the brain’s frontal pole (Talairach and Tournoux, 1988).

3) For the resting state scans, we eliminated the identified volumes together with the 1-back and the 2-forward volumes (to avoid spin history assumptions’ violations caused by movement; Power et al., 2012) from the frequency-filtered time series.

4) For the task-related scan, a trial with an onset within the window of three volumes starting with an identified motion-contaminated volume were discarded as events of interests, by adding them to a separate regressor that modelled across conditions all trials contaminated by fast head movement.

5) Participants were excluded from the dataset if more than 25% of the volumes (for resting state scans) or the trials (for task-related scans) were contaminated by fast head-movement. This led to the exclusion of 3 participants in the MD group, 1 in the FH group, and 1 in the HC group.

6) Finally, to ensure that group BOLD effects would not be attributable to movement, we compared and equated the three groups on the total number of removed trials and the mean relative translation distance after cleaning. This required excluding one participant in the MD group (i.e., with the maximum mean relative translation distance). For the task-related scans, the number of removed trials was equal across the three groups (*F* (2, 82) = 2.74, *p* = .07), as was the mean voxel-to-voxel displacement after cleaning (*F* (2, 82) = 2.57, *p* = .08). For the resting state scans the number of volumes contaminated by fast movement was larger in the MD group (*F* (2, 82) = 6.44, *p* < .01, and *F* (2, 82) = 4.19, *p* = .02), respectively), but after removing contaminated voxels there was no significant difference in average volume-wise displacement between the groups (*F* (2, 82) = 1.94, *p* = .15, and *F* (2, 82) = 1.63, *p* = .20), respectively).

7) For every participant the average variance in relative volume-to-volume displacement in the Z-direction, after discarding volumes with accessive head motion (see steps 2-4, above), was used as a regressor of no interest in the second level, random effects analyses of the task-related and resting state data (van Dijk et al., 2020).

The remaining number of participants was 28 in the HC group, 30 in the FH group and 28 in the MD group, as described in the Methods section of the paper.

## 2.4. Task-related fMRI analyses

Statistical analysis of the functional data was also performed using SPM 12 software (3) and MATLAB scripts (Mathworks, Natick, MA, USA, version 9.6).

*First-level Analysis*: For each individual data set, task-related modulations of BOLD signal were estimated with General Linear Models (GLM). In the statistical model the presentations of face pictures with a specific emotional valence (happy, neutral, and sad faces) were included as three independent regressor. Stimulus presentations for which a participant made an erroneous response were not modelled as separate events. The BOLD response was modelled by convoluting the onset of each stimulus presentation within a condition with a canonical Hemodynamic Response Function along with its temporal and dispersion derivatives. In addition to these regressors of interest, the six realignment parameters and the mean cerebrospinal fluid and white matter signal were added as variables of no interest to the statistical model. Low-frequency drift was filtered out with the standard 128 second high-pass filter and voxel autocorrelation was modelled with the standard first-order autoregressive model implemented in SPM12. For each of the regressors of interest the estimated weight in a voxel was related to the estimated session specific mean voxel signal, to compute a percent BOLD signal change. This resulted in 3 percent signal change (PSC) maps per participant, to be used as dependent data in the second level analysis.

*Second-level analysis:* For the 2nd level group analysis a mixed two-factor GLM model was created with as between-subject factor groups (3 levels: MD, FH, HC) and as within-subject factor emotional valence of the pictures (3 levels: happy, neutral, and sad faces). This resulted in 9 condition-specific regressors, organized in a 3 by 3 mixed model.

*Second-level analysis – Covariates***:** The following covariates were added as regressors of no interest to the 2nd level model: age, gender, nonverbal IQ, average volume-to-volume head displacement (see section 2.3-7), comorbidity, use of antidepressant medication, BDI-II scores, and BSI-PSDI scores. The last four regressors were entered in the analysis in a group-specific way. Not only are comorbidity (0 = no, 1 = yes) and medication status (0 = no antidepressant medication, 1 = taking antidepressant medication) only applicable to the MD group, but the BDI-II and PSDI scores are strongly correlated with the disease status of the experimental groups. Were these covariates defined across groups, they would be highly correlated with the effect of interest, namely differences in BOLD signal between the MD, FH and HC groups. Consequently, large portions of variation of interest would be regressed out of the analysis. To prevent this, (1) the group-specific covariate values were mean normalized within the participants of each group to an average value of zero, and (2) these normalized values were entered in group-specific regressors which contained the normalized covariate values for the participants within a specific group and zeros for the participants in the other groups. Consequently, the covariate values could only correct BOLD signal values within the specific group, while the average covariate level did not differ between the groups, as it was zero for each groups. This procedure yielded 8 group-specific regressors in the design matrix (3 BDI regressors, 3 PSDI regressors, 1 medication, and 1 comorbidity), in addition to the four already mentioned across group regressors (age, gender, nonverbal IQ, head motion).

*Definition of MDD vulnerability:* The central part in this study’s analysis was to detect voxels embodying MDD vulnerability. These are voxels whose task-related BOLD response does not differ between the MD group and the FH group, while being significantly different in the comparison of the MD vs. the HC group, as well as the comparison of the FH vs. the HC group. These voxels were identified in three staps: First, three pairwise group contrasts were computed, which pooled the three levels of the emotional valence factors, but looked for differences between the levels of the group factor: HC versus FH, HC versus MD, and FH versus MD. The three resulting F-statistic maps were thresholded at the False Discovery Rate (FDR) significance level α = .05, to correct for the multiple *F* tests computed across all the voxels in the image (Genovese ea., 2002; Nichols, 2012). In the second step, a conjunction map was created from two thresholded group contrasts, namely the HC versus FH map and the HC versus MD map. In this conjunction map all voxels that did not meet the FDR corrected threshold in one or both of the pairwise *F*-maps had a value of “0”, while all the voxels with a significant *F*-value in both pairwise *F*-maps had as their value the minimum of the two significant *F*-values. In the third step, a third group contrast, between the FH and MD groups, was used to exclusively mask the conjunction map at a lenient significance threshold of *p* = .001, uncorrected for multiple corrections. This means that all the voxels in the conjunction map with a value in the FH versus MD contrast that was higher than the critical *F*-value for p < .001 were set to the value “0”. As a result, voxels with a value higher than 0.0 were voxels that significantly differed in both the HC versus FH and HC versus MD contrasts, but did not significantly differ in the FH versus MD contrast. These were the voxels that indicated MDD vulnerability.

*ROI-based group analyses:* The Vulnerability analysis described above does not look into the effect that the emotional content of the faces had on the brain responses in the three groups. An emotional bias in the brain response of a particular vulnerability cluster would appear in the analysis of its PSC values as a significant interaction between the factors group and emotional content of the faces. To establish any such bias a follow-up ROI-based group analysis was performed in the IBM SPSS statistics (version 25) software package, after extracting the PSC averaged over the voxels in each vulnerability cluster in each of the participants. Separate values were obtained for the three emotional content conditions. For each cluster a 2-factor mixed model analysis was performed, with group (levels HC, FH and MD) as the between subject factor, emotional content of the faces (levels sad, neutral and happy) as the within subject factor, and with all 12 between-subjects’ covariates as described in section 2.4 above: age, gender, nonverbal IQ, average volume-to-volume head displacement (see section 2.3-7), and the within group normalized BDI scores for each group, the PSDI-scores for each group and the medication and the comorbidity scores for the MD group. A separate analysis was performed for each vulnerability cluster, at the statistical significance level α = 0.05. Because the sphericity estimate of the emotional content factor was consistently above ε > 0.70, the univariate test was chosen above the multivariate test (Field, 2018). Moreover, because in almost all cases ε > 0.75, the Huyn-Feldt correction was chosen over the Greenhouse-Geister correction (Field, 2018).

Repeating the statistical test for each cluster cumulates the chance of finding false positives in some of the tests performed. Applying the Bonferroni correction for multiple comparisons would reduce the α-level from 0.05 to 0.0017, which would render the sensitivity of the tests unacceptable. Therefore, we used the cumulative binomial coefficient as a safeguard against false positive results. Therefore, we only interpreted significant tests, if over all 29 tests, the chance of the observed or a higher number of significant tests was statistically unlikely, i.e., smaller than α = 0.05, under the null-hypothesis that there is 5% chance of observing a significant test in random data.

## 2.5. Functional connectivity analysis.

*Time series length.* In 6 participants 203 volumes (TR2.5s => 8.45833 min) were collected, instead of 153 volumes (6.375 min). These data were collected in the beginning of the study, and included patients from all three groups. We used all the images acquired, i.e., the six datasets were not shortened to match the shorter timeseries data of the other participants. This choice was based in the realization that due to the head motion correction (see section S2.3, above) the lengths of the timeseries entered in the analyses would anyway differ (somewhat) across participants. We performed a statistical comparison of the number of volumes entering the analyses across the three groups and found no significant differences (F (2, 106) = 0.468, p = .628)

*Seed definition*. For the investigation of differences in the interactions between clusters of activity change indicative of MDD vulnerability, the activation clusters were grouped into 7 seeds, each with a left and right hemisphere homologue, yielding 14 seeds. The relationship between the seeds clusters and the vulnerability clusters is indicated in Supplement table S2.

*Seed-by-seed correlation matrix*: For each participant a seed-by-seed correlation matrix was computed. Seed time courses were obtained by averaging the voxel-time courses within each seed and grey matter mask. A Fischer-Z transformation improved the normality distribution of the correlation coefﬁcients.

*Second level correlation matrix analysis*: The correlation matrices per participant were subjected to a seed-by-seed specific statistical analysis, in which for every pair of seeds a 3 factor ANOVA was performed, with group is a between subject factor with 2 levels (either FH versus HC or MD versus HC), and the seeds as two within subject factors, each with to levels (i.e., left and right hemisphere).

To reduce the amount of unaccounted variance in the design, the following measures were added as regressors of no interest to the statistical model: age, gender, nonverbal IQ, head motion translation variance (see section 2.3-7), comorbidity, use of antidepressant medication, BDI-II scores, and BSI-PSDI scores. These were again added in a group-specific manner, as described above (Section S2.4, above).

*Multiple comparisons correction*: Because our research question does not involve any laterality effects, only the main effect of age group will be presented in the results section. The direction of the effect was inferred from the mean correlation values in each group. Since there were 7 seeds, the total number of tests performed was (7² - 7)/2 = 21 tests. Each of these tests was evaluated for significance at the uncorrected α-level of 0.05. To guard against the cumulating chance of finding false positives, the cumulative binomial coefficient assessed the likelihood that the observed number of positive tests over the repeated analyses was attributable to false positives. This test assesses what the probability is of obtaining an equal or higher number of hits (i.e., significant tests) than the actually observed hit-rate given the number of tests performed and a random hit rate equal to α (i.e., 0.05). These probability checks were computed separately for the FH-HC and the MD-HC analyses. Single seed-by-seed effects at α = 0.05 were only interpreted as meaningful if the cumulative binomial probability of the observed single effects on similar tests for all the seed pairs was less than 0.05 – i.e., if the number of positive tests was significantly unlikely under the null hypothesis that there were no group difference in the data.

# 3. Supplementary Figures & Tables

**Supplementary Table S1.** Demographic and clinical data of the MD patient group.

| **Age** | **Gender** | **Axis I Comorbidity** | **Current Medications** | **Med Type** |
| --- | --- | --- | --- | --- |
| 22 | F |  |  |  |
| 31 | F | PTSD | Venlafaxine | SNRI |
| 59 | F |  |  |  |
| 49 | M | Social Phobia |  |  |
| 34 | F |  |  |  |
| 18 | F |  | Citalopram | SSRI |
| 25 | F | Social Phobia | Sertraline | SSRI |
| 49 | F |  | Paroxetine | SSRI |
| 20 | M | PTSD |  |  |
| 52 | M |  |  |  |
| 31 | F |  | Nortriptyline | TCA |
| 23 | F | Specific Phobia | Bupropion | NDRI |
| 52 | F | Specific Phobia | Citalopram | SSRI |
| 47 | M |  | Venlafaxine, Seroquel | SNRI, Antipsychotic |
| 44 | M |  | Citalopram, Mirtazapine | SSRI, TetraCA |
| 25 | F |  | Agomelatine | Melatonergic |
| 36 | F |  |  |  |
| 39 | F | Social Phobia, GAD |  |  |
| 52 | M |  |  |  |
| 44 | M | GAD | Paroxetine | SSRI |
| 42 | F | Panic Disorder with agoraphobia | Citalopram | SSRI |
| 54 | F |  |  |  |
| 55 | F | PTSD | Citalopram | SSRI |
| 49 | F |  |  |  |
| 37 | M | Specific Phobia, Social Phobia |  |  |
| 38 | F | Social Phobia |  |  |
| 44 | F |  |  |  |
| 36 | F | Social Phobia |  |  |
| 38 | F |  | Escitalopram | SSRI |
| 22 | F |  |  |  |
| 19 | F | GAD, Panic with agoraphobia, ADHD/ADD | Citalopram | SSRI |
| 34 | M | Panic Disorder with agoraphobia, Social Phobia | Escitalopram | SSRI |
| 43 | M | Social Phobia |  |  |
| 54 | F | Agoraphobia |  |  |
| 44 | F | Panic disorder, OCD | Fluvoxamine | SSRI |

**Abbreviations:** PTSD (Post Traumatic Stress Disorder); GAD (Generalized Anxiety Disorder); ADHD/ADD (Attention Deficit Hyperactivity Disorder); OCD (Obsessive Compulsive Disorder); SNRI (Serotonin Norepinephrine Reuptake Inhibitor); SSRI (Selective Serotonin Reuptake Inhibitor); TCA (Tricyclic Antidepressant); NDRI (Norepinephrine Dopamine Reuptake Inhibitor); TetraCA (Tetracyclic Antidepressant).

**Supplementary table S2**: All 64 voxel clusters with different activity in the healthy control (HC) compared to the family history of depression (FH) and major depression (MD) participants, while activity in the latter two groups did not differ. Clusters are order posterior to anterior based on y-coordinates. ID number refers to the manuscript—clusters with no ID number are not among the 29 clusters discussed in the manuscript.

| MD vulnerability cluster description | | | | | | | | |  | Functional characteristics | | | | | | | | | | | | | |
| --- | --- | --- | --- | --- | --- | --- | --- | --- | --- | --- | --- | --- | --- | --- | --- | --- | --- | --- | --- | --- | --- | --- | --- |
|  |  |  |  |  |  |  |  |  |  |  |  |  |  |  |  | Percent signal change^d^ | | | | | | | |
|  |  |  |  | Center of mass (mm) | | |  |  |  | Relation | |  | Direction | |  | HC | |  | FH | |  | MD | |
| ID | Location ^a^ | Side |  | x | y | z |  | N vxls |  | to task cortex ^b^ | |  | of change ^c^ | |  | M | SD |  | M | SD |  | M | SD |
|  | Dorsal occipital pole | Left |  | -19 | -96 | 8 |  | 2 |  | task pos. |  |  | pos. |  |  | 0.52 | 0.27 |  | 0.72 | 0.37 |  | 0.62 | 0.33 |
|  | Dorsal occipital pole | Left |  | -11 | -92 | 37 |  | 2 |  | In between | |  |  | neg. |  | 0.44 | 0.67 |  | -0.05 | 0.46 |  | 0.01 | 0.62 |
|  | Calcarine sulcus | Left |  | -10 | -91 | -1 |  | 4 |  | task pos. |  |  |  | neg. |  | 0.99 | 0.57 |  | 0.73 | 0.49 |  | 0.71 | 0.45 |
| 2 | Lingual gyrus | Bilateral |  | 4 | -91 | -9 |  | 16 |  | task pos. |  |  |  | neg. |  | 1.43 | 0.64 |  | 1.09 | 0.59 |  | 1.08 | 0.56 |
|  | Dorsal occipital cortex | Right |  | 26 | -90 | 12 |  | 1 |  | task pos. |  |  | pos. |  |  | 0.39 | 0.32 |  | 0.59 | 0.35 |  | 0.56 | 0.52 |
| 6 | Collateral sulcus | Right |  | 25 | -90 | -11 |  | 12 |  | task pos. |  |  | pos. |  |  | 0.42 | 0.42 |  | 0.72 | 0.44 |  | 0.66 | 0.39 |
| 13 | Ventral Intraparietal sulcus | Left |  | -33 | -89 | 26 |  | 7 |  | task pos. |  |  |  | neg. |  | -0.02 | 0.34 |  | -0.20 | 0.29 |  | -0.15 | 0.36 |
|  | Ventral occipital cortex | Left |  | -26 | -89 | -14 |  | 2 |  | task pos. |  |  | pos. |  |  | 0.40 | 0.35 |  | 0.66 | 0.43 |  | 0.58 | 0.47 |
|  | Lateral occipital cortex | Left |  | -46 | -83 | 6 |  | 2 |  | task pos. |  |  | pos. |  |  | 0.15 | 0.26 |  | 0.30 | 0.41 |  | 0.30 | 0.37 |
|  | Ventral occipital cortex | Left |  | -31 | -82 | -25 |  | 2 |  | task pos. |  |  |  | neg. |  | 0.42 | 0.59 |  | 0.18 | 0.47 |  | 0.16 | 0.32 |
|  | Ventral occipital cortex | Left |  | -34 | -79 | -22 |  | 2 |  | task pos. |  |  |  | neg. |  | 0.38 | 0.33 |  | 0.16 | 0.40 |  | 0.15 | 0.33 |
| 3 | Lingual gyrus | Left |  | -7 | -81 | -16 |  | 25 |  | task pos. |  |  |  | neg. |  | 0.88 | 0.55 |  | 0.56 | 0.52 |  | 0.55 | 0.39 |
| 1 | Calcarine sulcus | Bilateral |  | 8 | -80 | 5 |  | 185 |  | task pos. |  |  |  | neg. |  | 0.62 | 0.38 |  | 0.34 | 0.26 |  | 0.30 | 0.32 |
| 14 | Ventral Intraparietal sulcus | Right |  | 40 | -78 | 33 |  | 6 |  |  | de-activ. |  |  | neg. |  | -0.18 | 0.20 |  | -0.34 | 0.41 |  | -0.28 | 0.23 |
|  | Ventral Intraparietal sulcus | Right |  | 24 | -78 | 24 |  | 4 |  | task pos. |  |  | pos. |  |  | 0.01 | 0.16 |  | 0.13 | 0.22 |  | 0.10 | 0.23 |
| 4 | Lingual gyrus | Right |  | 18 | -77 | -22 |  | 15 |  | task pos. |  |  |  | neg. |  | 0.26 | 0.41 |  | 0.10 | 0.22 |  | 0.11 | 0.20 |
| 7 | Fusiform gyrus posterior | Left |  | -39 | -77 | -17 |  | 14 |  | task pos. |  |  | pos. |  |  | 0.77 | 0.50 |  | 1.23 | 0.72 |  | 1.23 | 0.57 |
|  | Parietal-occipital sulcus | Right |  | 18 | -75 | 31 |  | 2 |  |  | de-activ. |  |  | neg. |  | -0.05 | 0.20 |  | -0.15 | 0.26 |  | -0.16 | 0.22 |
|  | Calcarine sulcus | Right |  | 12 | -74 | 14 |  | 1 |  | task pos. |  |  |  | neg. |  | 0.29 | 0.43 |  | 0.12 | 0.32 |  | 0.09 | 0.34 |
| 8 | Lateral occipital cortex | Right |  | 53 | -73 | 0 |  | 60 |  | task pos. |  |  | pos. |  |  | 0.26 | 0.26 |  | 0.48 | 0.32 |  | 0.51 | 0.35 |
|  | Fusiform gyrus, lateral tip | Right |  | 41 | -72 | -19 |  | 3 |  | task pos. |  |  |  | neg. |  | 1.35 | 1.07 |  | 1.01 | 0.65 |  | 0.94 | 0.66 |
| 5 | Lingual gyrus anterior | Right |  | 5 | -65 | 3 |  | 6 |  | task pos. |  |  |  | neg. |  | 0.41 | 0.50 |  | 0.21 | 0.37 |  | 0.19 | 0.29 |
|  | Fusiform gyrus, lateral tip | Left |  | -41 | -65 | -24 |  | 5 |  | task pos. |  |  |  | neg. |  | 0.50 | 0.37 |  | 0.31 | 0.35 |  | 0.35 | 0.39 |
| 15 | Intraparietal sulcus | Left |  | -40 | -63 | 37 |  | 13 |  |  | de-activ. |  |  | neg. |  | 0.00 | 0.19 |  | -0.10 | 0.19 |  | -0.11 | 0.16 |
| 16 | Intraparietal sulcus | Right |  | 35 | -61 | 29 |  | 12 |  |  |  |  | pos. |  |  | -0.05 | 0.11 |  | 0.04 | 0.16 |  | 0.04 | 0.14 |
| 17 | Intraparietal sulcus | Left |  | -43 | -53 | 51 |  | 8 |  | task pos. |  |  | pos. |  |  | -0.06 | 0.22 |  | 0.07 | 0.21 |  | 0.09 | 0.24 |
| 9 | Inferior temporal sulcus | Left |  | -61 | -52 | -13 |  | 16 |  |  | de-activ. |  | pos. |  |  | -0.24 | 0.22 |  | -0.12 | 0.19 |  | -0.09 | 0.25 |
| 10 | Inferior temporal sulcus | Right |  | 56 | -52 | -5 |  | 9 |  |  | de-activ. |  | pos. |  |  | -0.12 | 0.15 |  | -0.02 | 0.17 |  | 0.00 | 0.18 |
| Prefrontal (and insular) | |  |  |  |  |  |  |  |  |  |  |  |  |  |  |  |  |  |  |  |  |  |  |
|  | Inferior temporal gyrus | Left |  | -46 | -50 | -32 |  | 1 |  | task pos. |  |  |  | neg. |  | 0.21 | 0.35 |  | 0.06 | 0.21 |  | 0.05 | 0.20 |
|  | Inferior temporal gyrus | Left |  | -45 | -49 | -36 |  | 3 |  | task pos. |  |  |  | neg. |  | 0.11 | 0.29 |  | -0.08 | 0.19 |  | -0.05 | 0.17 |
| 11 | Fusiform gyrus | Left |  | -25 | -45 | -19 |  | 6 |  |  | de-activ. |  | pos. |  |  | 0.00 | 0.21 |  | 0.10 | 0.20 |  | 0.11 | 0.26 |
| 20 | Intraparietal sulcus | Left |  | -35 | -10 | 14 |  | 19 |  | task pos. |  |  | pos. |  |  | 0.09 | 0.09 |  | 0.16 | 0.16 |  | 0.17 | 0.14 |
|  | Inferior frontal sulcus | Left |  | -58 | -36 | -16 |  | 1 |  |  | de-activ. |  | pos. |  |  | -0.08 | 0.19 |  | 0.03 | 0.21 |  | 0.03 | 0.22 |
|  | Postcentral/intraparietal sulcus | Right |  | 37 | -36 | 53 |  | 4 |  |  | de-activ. |  | pos. |  |  | -0.02 | 0.17 |  | 0.08 | 0.17 |  | 0.07 | 0.16 |
| 12 | Fusiform gyrus anterior | Right |  | 17 | -35 | -23 |  | 14 |  |  | de-activ. |  | pos. |  |  | -0.05 | 0.16 |  | 0.06 | 0.14 |  | 0.05 | 0.17 |
| 20 | Postcentral sulcus | Left |  | -37 | -32 | 43 |  | 5 |  | task pos. |  |  | pos. |  |  | 0.10 | 0.13 |  | 0.18 | 0.18 |  | 0.19 | 0.17 |
| 18 | Postcentral sulcus | Right |  | 40 | -28 | 46 |  | 1 |  | task pos. |  |  | pos. |  |  | 0.10 | 0.15 |  | 0.15 | 0.14 |  | 0.16 | 0.13 |
| 18 | Intraparietal sulcus | Right |  | 45 | -27 | 43 |  | 10 |  | task pos. |  |  | pos. |  |  | 0.15 | 0.11 |  | 0.21 | 0.14 |  | 0.23 | 0.14 |
| 20 | Postcentral sulcus | Left |  | -36 | -24 | 50 |  | 1 |  | task pos. |  |  | pos. |  |  | 0.12 | 0.17 |  | 0.19 | 0.21 |  | 0.23 | 0.19 |
| 19 | Postcentral/intraparietal sulcus | Right |  | 58 | -21 | 47 |  | 101 |  | task pos. |  |  | pos. |  |  | 0.10 | 0.16 |  | 0.26 | 0.22 |  | 0.27 | 0.20 |
| 21 | Postcentral/intraparietal sulcus | Left |  | -53 | -18 | 43 |  | 44 |  | task pos. |  |  | pos. |  |  | 0.04 | 0.22 |  | 0.17 | 0.14 |  | 0.19 | 0.18 |
|  | Supramarginal gyrus | Right |  | 60 | -18 | 32 |  | 1 |  |  | de-activ. |  | pos. |  |  | 0.01 | 0.16 |  | 0.09 | 0.20 |  | 0.08 | 0.21 |
|  | Superior frontal/precentral sulcus | Left |  | -30 | -18 | 62 |  | 1 |  |  | de-activ. |  | pos. |  |  | -0.02 | 0.18 |  | 0.08 | 0.20 |  | 0.10 | 0.18 |
|  | Postcentral gyrus | Left |  | -46 | -18 | 64 |  | 1 |  | task pos. |  |  | pos. |  |  | 0.34 | 0.42 |  | 0.64 | 0.67 |  | 0.70 | 0.45 |
|  | Postcentral gyrus | Left |  | -54 | -17 | 57 |  | 5 |  | task pos. |  |  | pos. |  |  | 0.24 | 0.39 |  | 0.48 | 0.41 |  | 0.55 | 0.42 |
|  | Precentral gyrus | Right |  | 55 | -13 | 55 |  | 4 |  | task pos. |  |  | pos. |  |  | 0.06 | 0.38 |  | 0.28 | 0.48 |  | 0.29 | 0.34 |
| 27 | Ventral thalamus | Left |  | -14 | -12 | 2 |  | 6 |  | task pos. |  |  |  | neg. |  | 0.08 | 0.10 |  | 0.02 | 0.09 |  | 0.02 | 0.10 |
| 22 | Superior frontal/precentral sulcus | Right |  | 41 | -5 | 53 |  | 22 |  | task pos. |  |  | pos. |  |  | 0.05 | 0.18 |  | 0.14 | 0.17 |  | 0.16 | 0.17 |
| 28 | Amygdala | Right |  | 17 | -5 | -15 |  | 11 |  | task pos. |  |  | pos. |  |  | 0.15 | 0.26 |  | 0.30 | 0.41 |  | 0.30 | 0.37 |
|  | Caudate body | Left |  | -22 | -2 | 22 |  | 1 |  |  | de-activ. |  |  | neg. |  | 0.05 | 0.11 |  | -0.01 | 0.11 |  | -0.01 | 0.11 |
| 23 | Insular cortex | Left |  | -34 | 5 | 8 |  | 10 |  | task pos. |  |  |  | neg. |  | 0.02 | 0.12 |  | -0.04 | 0.13 |  | -0.04 | 0.13 |
| 24 | Middle cingulate cortex | Left |  | -9 | 8 | 38 |  | 4 |  | task pos. |  |  |  | neg. |  | 0.15 | 0.15 |  | 0.07 | 0.16 |  | 0.05 | 0.18 |
| 24 | Anterior middle cingulate cortex | Left |  | -6 | 12 | 38 |  | 1 |  | task pos. |  |  |  | neg. |  | 0.17 | 0.15 |  | 0.10 | 0.21 |  | 0.10 | 0.18 |
| 24 | Cingulate sulcus (aCMA) | Left |  | -7 | 13 | 36 |  | 11 |  | task pos. |  |  |  | neg. |  | 0.17 | 0.13 |  | 0.11 | 0.12 |  | 0.12 | 0.12 |
| 29 | Ventral striatum | Right |  | 14 | 19 | 0 |  | 14 |  |  |  |  |  | neg. |  | 0.03 | 0.13 |  | -0.08 | 0.12 |  | -0.05 | 0.12 |
|  | Precentral sulcus | Left |  | -31 | 20 | 33 |  | 2 |  |  | de-activ. |  |  | neg. |  | 0.02 | 0.15 |  | -0.04 | 0.12 |  | -0.07 | 0.14 |
| 25 | Middle frontal gyrus | Left |  | -34 | 33 | 41 |  | 16 |  |  | de-activ. |  |  | neg. |  | -0.02 | 0.16 |  | -0.12 | 0.19 |  | -0.13 | 0.16 |
|  | Anterior middle cingulate cortex | Left |  | -2 | 36 | 18 |  | 1 |  |  | de-activ. |  |  | neg. |  | -0.02 | 0.16 |  | -0.10 | 0.15 |  | -0.08 | 0.18 |
|  | Anterior superior frontal sulcus | Left |  | -20 | 38 | 40 |  | 4 |  |  | de-activ. |  | pos. |  |  | -0.10 | 0.17 |  | 0.00 | 0.19 |  | -0.03 | 0.17 |
| 26 | Frontal Pole | Right |  | 25 | 54 | -1 |  | 2 |  |  | de-activ. |  |  | neg. |  | -0.02 | 0.17 |  | -0.11 | 0.17 |  | -0.11 | 0.22 |
|  | Frontal Pole | Left |  | -34 | 56 | -5 |  | 5 |  |  | de-activ. |  | pos. |  |  | -0.10 | 0.21 |  | 0.05 | 0.21 |  | 0.01 | 0.24 |
| 26 | Frontal Pole | Right |  | 20 | 64 | 12 |  | 1 |  |  | de-activ. |  |  | neg. |  | 0.04 | 0.22 |  | -0.05 | 0.21 |  | -0.07 | 0.22 |
| 26 | Frontal Pole | Right |  | 25 | 65 | 1 |  | 20 |  |  |  |  |  | neg. |  | 0.31 | 0.24 |  | 0.13 | 0.14 |  | 0.22 | 0.18 |

Note. Clusters are roughly listen in the order of their anterior-dorsal position (Y-dimension), and group according to functional properties.

^a^ For specific anatomical locations of visual areas we followed (Stiers et al., 2006).

^b^ Location of vulnerability cluster in or near task-modulated cortex, i.e., either task-activated (“task pos.”) zones or task-de-activated (“de-activ.”) zones in the HC group.

^c^ Activity level of FH and MD groups is either higher (pos.) or lower (neg.) than in the HC group.

^d^ Percent signal change across all trials (i.e., regardless of affective expression of the face stimuli), in 10% most responsive voxels in each cluster, or all voxels for clusters with less than 10 voxels.

Abbreviations: vxls = voxels; N = number; M = mean; SD = standard deviation.

**Supplementary Table S3.** Explanation of building of functional connectivity seeds from the vulnerability clusters observed in Table 2.

|  | Seed regions | |  |  | Vulnerability clusters |  |
| --- | --- | --- | --- | --- | --- | --- |
| Name | hemisphere | Anatomical site |  | Cluster.ID | Definition | NOfVoxels |
| V1/V2 | left | calcarine sulcus |  | 1 | sphere with center [-2, -86, 8] and radius 8 mm | 48 |
| V1/V2 | right |  |  | 1 | sphere with center [12, -82, 8] and radius 8 mm | 57 |
| Lingual G. | left | lingual gyrus |  | 2 | sphere with center [0, -92, -13] and radius 8 mm | 8 |
|  |  |  |  | 3 | all voxels in cluster | 25 |
| Lingual G. | right |  |  | 2 | sphere with center [8, -88, -5] and radius 8 mm | 8 |
|  |  |  |  | 4, 5 | all voxels in clusters | 21 |
| Vvis pos. | left | posterior ventral visual cortex |  | 7 | all voxels in cluster | 14 |
| Vvis pos. | right |  |  | 6 | all voxels in cluster | 12 |
| LOC | left | lateral occipital cortex |  | -8* | sphere with center [-53, -73, 0] and radius 3 mm | 16 |
| LOC | right |  |  | 8 | all voxels in cluster | 60 |
| Vvis ant. | left | anterior ventral visual cortex |  | 9, 11 | all voxels in clusters | 22 |
| Vvis ant. | right |  |  | 10, 12 | all voxels in clusters | 23 |
| vIPS | left | ventral intraparietal sulcus |  | 13, 15 | all voxels in clusters | 20 |
| vIPS | right |  |  | 14 | all voxels in cluster | 6 |
| dIPS/PCS | left | dorsal intraparietal /postcentral sulcus |  | 17, 20, 21 | all voxels in clusters | 71 |
| dIPS/PCS | right |  |  | 18, 19 | all voxels in clusters | 111 |
| BNM-CH04 | left | basal nucleus of Meynert - CH 04 |  | -- | all voxels in mask | 32 |
| BNM-CH04 | right |  |  | -- | all voxels in mask | 27 |

Note: * As there was no LOC activation cluster in right hemisphere, the seed was created as the homologue of the left hemisphere cluster.

# References

Nichols, T. E. (2012). Multiple testing corrections, nonparametric methods, and random field theory. NeuroImage, 62(2), 811-815. https://doi.org/10.1016/j.neuroimage.2012.04.014

Genovese, C.R., Lazar, N.A., and Nicols, T. (2002). Thresholding of statistical maps in functional neuroimaging using the false discovery rate. NeuroImage 15, 870–878.

Field, A. P. (2018). Discovering statistics using IBM SPSS statistics (5th ed.). SAGE Publications.

Stevens, J. (2002). Applied multivariate statistics for the social sciences (4th ed.). L. Erlbaum.

Talairach J., Tournoux P. Co-Planar Stereotaxic Atlas of the Human Brain: 3-Dimensional Proportional System: An Approach to Cerebral Imaging. Thieme; Stuttgart, Germany: 1988.
